# Shared Metabolic Profile of Caffeine in Parkinsonian Disorders

**DOI:** 10.1002/mds.28068

**Published:** 2020-05-01

**Authors:** Haruka Takeshige‐Amano, Shinji Saiki, Motoki Fujimaki, Shin‐Ichi Ueno, Yuanzhe Li, Taku Hatano, Kei‐Ichi Ishikawa, Yutaka Oji, Akio Mori, Ayami Okuzumi, Taiji Tsunemi, Kensuke Daida, Yuta Ishiguro, Yoko Imamichi, Hisayoshi Nanmo, Shuko Nojiri, Manabu Funayama, Nobutaka Hattori

**Affiliations:** ^1^ Department of Neurology Juntendo University School of Medicine Tokyo Japan; ^2^ Mathematical Science Unit, Graduate School of Engineering Science Yokohama National University Kanagawa Japan; ^3^ Medical Technology Innovation Center Juntendo University Tokyo Japan; ^4^ Research Institute of Diseases of Old Age, Graduate School of Medicine Juntendo University Tokyo Japan

**Keywords:** caffeine, caffeine metabolites, multiple system atrophy, Parkinson's disease, progressive supranuclear palsy

## Abstract

**Objective:**

The objective of this study was to determine comprehensive metabolic changes of caffeine in the serum of patients with parkinsonian disorders including Parkinson's disease (PD), progressive supranuclear palsy (PSP), and multiple system atrophy (MSA) and to compare this with healthy control serum.

**Methods:**

Serum levels of caffeine and its 11 downstream metabolites from independent double cohorts consisting of PD (n = 111, 160), PSP (n = 30, 19), MSA (n = 23, 17), and healthy controls (n = 43, 31) were examined by liquid chromatography–mass spectrometry. The association of each metabolite with clinical parameters and medication was investigated. Mutations in caffeine‐associated genes were investigated by direct sequencing.

**Results:**

A total of 9 metabolites detected in more than 50% of participants in both cohorts were decreased in 3 parkinsonian disorders compared with healthy controls without any significant association with age at sampling, sex, or disease severity (Hoehn and Yahr stage and Unified Parkinson's Disease Rating Scale motor section) in PD, and levodopa dose or levodopa equivalent dose in PSP and MSA. Of the 9 detected metabolites, 8 in PD, 5 in PSP, and 3 in MSA were significantly decreased in both cohorts even after normalizing to daily caffeine consumption. No significant genetic variations in *CYP1A2* or *CYP2E1* were detected when compared with controls.

**Conclusion:**

Serum caffeine metabolic profiles in 3 parkinsonian diseases show a high level of overlap, indicative of a common potential mechanism such as caffeine malabsorption from the small intestine, hypermetabolism, increased clearance of caffeine, and/or reduced caffeine consumption. © 2020 The Authors. *Movement Disorders* published by Wiley Periodicals, Inc. on behalf of International Parkinson and Movement Disorder Society.

Parkinson's disease (PD) is a common, progressive, neurodegenerative disease characterized by motor symptoms (including akinesia, resting tremor, and rigidity) as well as nonmotor symptoms.^1^, ^2^, ^3^ Progressive supranuclear palsy (PSP) and multiple system atrophy (MSA) exhibit overlapping clinical motor symptoms of PD, which can cause difficulties in differential diagnosis. Although each disease has disease‐specific pathological features such as neuronal Lewy bodies/neurites (PD), glial cytoplasmic inclusions (MSA), and neuronal tau accumulation (PSP), progressive nigral degeneration is a common manifestation of all these diseases, implying shared parkinsonian motor symptoms.^4^, ^5^, ^6^, ^7^

Caffeine is the most common psychostimulant and has attracted attention for its neuroprotective effects via inhibition of lipid peroxidation and reduction of reactive oxygen species production.^8^ The microbiome was changed by the administration of caffeine, and this was associated with the attenuation of inflammation.^9^, ^10^, ^11^ Studies using 1‐methyl‐4‐phenyl‐1,2,3,6‐tetrahydropyridine animal models revealed its neuroprotective effects by protecting against the loss of striatal dopaminergic neurons by adenosine A_2A_ receptor blockade.^12^ The *ADORA2A* gene encodes adenosine A_2A_ receptors in dopaminergic neurons. Although A_2A_ receptor mRNA was increased in the putamen of patients with PD and dyskinesia,^13^ the interactions of its polymorphisms and PD risk or their coffee consumption are still controversial,^14^, ^15^, ^16^, ^17^ and the differences in its frequency between PD and controls were not detected in our previous study.^18^ Several epidemiological studies have shown risk reduction for developing PD, especially in males.^19^, ^20^, ^21^, ^22^, ^23^ After the onset of PD, patients with de novo PD and a higher caffeine intake suffer less disease progression when compared with those with a reduced intake.^24^ Although a daily caffeine intake of 200 mg twice a day was beneficial for motor symptoms in a short‐term study of patients with PD,^25^ this result was not proven by a subsequent multicenter, long‐term trial.^26^ Furthermore, based on previous studies showing decreased caffeine levels in the serum/plasma of patients with PD,^27^, ^28^ we recently reported that serum caffeine and its 11 downstream metabolites were uniformly decreased without associations to disease severity or daily caffeine consumption amount, suggesting underlining pharmacokinetic differences in PD patients, such as malabsorption of caffeine.^18^ The neuroprotective effect of caffeine has been suggested in other neurodegenerative diseases, although comprehensive metabolic changes of caffeine in patients with MSA or PSP have not been reported.^8^

## Methods

### Participants

In the current study, we established the first cohort by random selection of patients with each disease and healthy controls (HCs) as a pilot study. After an analysis of the first cohort, we set up a second cohort to minimize the differences of age, sex, and daily caffeine consumption among the 4 groups. Participant characteristics in both the first and second cohorts are shown in Table 1. The first cohort included 111 patients with PD, 30 with PSP, 23 with MSA, and 43 HCs. In the second cohort, there were 160 patients with PD, 19 with PSP, 17 with MSA, and 31 HCs. All patients had no apparent family history of each disease and had been treated at Juntendo University Hospital (Tokyo, Japan). All HCs were recruited from spouses of patients, patients with hypertension or dyslipidemia treated with medication, and hospital/laboratory staff by poster advertisements in practice waiting rooms; were free from any neurodegenerative disease; and used the same writing consent and explanation forms during the same period as patients. Two HC participants in each cohort had a relative with PD or parkinsonian syndrome. PD was diagnosed according to the Movement Disorder Society Clinical Diagnostic Criteria for Parkinson's disease.^2^ PSP was diagnosed as possible or probable according to the Movement Disorder Society Criteria for Clinical Diagnosis of Progressive Supranuclear Palsy.^29^ MSA was diagnosed as possible or probable based on the consensus statement on the diagnosis of MSA by Gilman and colleagues.^30^ Hoehn and Yahr (H&Y) stages and Movement Disorder Society Unified Parkinson's Disease Rating Scale motor section (MDS‐UPDRS Part III) scores were defined during the *on* phase because most patients were examined at outpatient clinics.^31^ Levodopa equivalent doses (LEDs) were calculated based on a previous report.^32^ All participants, including HCs, had no history of tumor/cancer, aspiration pneumonia, gastrointestinal diseases, liver or renal dysfunction detected by conventional blood chemistries, or collagen vascular diseases.

**Table 1:** Participant characteristics in the first cohort

Characteristic	PD	PSP	*P* Value vs. PD	*P V*alue vs. HCs	MSA	*P* Value vs. PD	*P* Value vs. HCs	HC	*P* Value vs. PD
Number	111	30			23			43	
		Probable: 29 Possible: 1			Probable: 15 Possible: 8				
Sex, male:female	57:54	16:14	0.847^a^	0.244^a^	7:16	0.0676^a^	0.464^a^	17:26	0.188^a^
Age, y, mean (SD)	67.1 (9.99)	72.9 (6.44)	0.0187^b^	0.0022^b^	62.5 (9.07)	0.136^b^	0.977^b^	60.0 (14.8)	0.0466^b^
Daily caffeine consumption, mg/day, mean (SD)	100 (78.4)	65.3 (59.8)	0.0876^b^	<0.0001^b^	102 (80.6)	0.996^b^	0.0740^b^	150 (84.6)	0.0014^b^
Constipation, %	72.1	55.2	0.0809^a^	0.0246^a^	86.4	0.160^a^	<0.0001^a^	28.2	<0.0001
(Missing)	(0)	(1)			(1)			(4)	
Current smoker, %	4.67	0.00	0.228^a^	0.0112^a^	4.35	0.946^a^	0.101^a^	19.1	0.0052
(Missing)	(4)	(0)			(0)			(1)	
Disease duration, y, mean (SD)	6.34 (5.58)	4.70 (2.79)	0.523^b^		3.26 (1.57)	0.0187^b^			
LED, mg, mean (SD)	582 (358)	589 (328)	0.984^b^		454 (463)	0.0955^b^			
Levodopa, mg, mean (SD)	367 (241)	513 (275)	0.0285^b^		350 (309)	0.934^b^			

Total caffeine intake was calculated using the Food Society Commission of Japan guidelines. Caffeine content was assumed to be 60 mg per cup of coffee, 30 mg per cup of tea, and 20 mg per cup of green tea.

a *P* value obtained by chi‐square test.

b *P* value obtained by Steel‐Dwass test.

Abbreviations: PD, Parkinson's disease; PSP, progressive supranuclear palsy; HC, heathy controls; MSA, multiple system atrophy; SD, standard deviation; LED, levodopa equivalent dose.

### Caffeine Exposure Ascertainment

We used original self‐administered questionnaires to obtain the current caffeine consumption amount. In the questionnaire, we asked what types of drinks they have and the mean amount of each drink they have per day. Of note, the questionnaire reflects the actual caffeine intake as previously reported.^33^ Caffeine concentration was assessed as 60 mg per cup of coffee, 30 mg per cup of tea, and 20 mg per cup of green tea using the Food Society Commission of Japan guidelines.^34^ We referred to product information for energy drinks or carbonated drinks containing caffeine. Decaffeinated beverages were not included in this study.

### Standard Protocol Approvals, Registrations, and Patient Consents

The study protocol complied with the Declaration of Helsinki and was approved by the ethics committee of Juntendo University (no. 2015101). Written informed consent was provided by all participants.

### Data Availability Statement

All data, including clinical characteristics and scales and experimental data (metabolites and genomic DNAs), will be provided in Microsoft Excel format upon request from the corresponding author (S.S.).

### Sample Collection

Venous blood samples for laboratory analysis were collected between 9:00 am and 12:00 pm at the outpatient department of Juntendo University Hospital. All participants were only allowed to have water and medicines from 0:00 am until sampling to exclude the effect of caffeine intake immediately before the sampling, as the half‐life of caffeine is 5 to 6 hours, and the trough caffeine concentration is steady.^33^, ^35^ Compliance with this requirement was assessed by questioning before sampling. Serum samples were obtained using 8 mL INSEPACK tubes (Sekisui Medical, Tokyo, Japan) followed by 2 to 3 inversions and then stored at −80°C. Samples were rested for 30 to 60 minutes at 4°C followed by centrifugation for 15 minutes at 1710*g*. Blood samples from the HCs were also collected, stored, and processed under the identical conditions to those for the samples from the parkinsonian patients.

### Sample Preparation

To measure levels of caffeine and its 11 downstream metabolites (theophylline, theobromine, paraxanthine, 1,7‐dimethyluric acid, 1,3,7‐trimethyluric acid, 1‐methylxanthine, 3‐methylxanthine, 7‐methylxanthine, and 1,3‐dimethyluric acid, 3,7–dimethyluric acid, and 5‐acetylamino‐6‐formytamino‐3‐methyluracil [AAMU]; Supplementary Fig. 1), metabolomic analysis was performed based on a previous study.^36^ Briefly, 100 μL of serum samples were added to 200 μL of internal standards: caffeine‐(trimethyl‐^13^C_3_); 3‐methylxanthine‐2,4,5,6‐^13^C_4_, 1,3,9‐^15^N_3_; and 7‐methylxanthine‐2,4,5,6‐^13^C_4_, 1,3‐^15^N_2_ (all purchased from Sigma‐Aldrich, St. Louis, MO). Solutions were centrifuged at 1,710 g for 5 minutes at 4°C, and 100 μL of the upper aqueous layer was homogenized with 200 μL of methanol followed by nitrogen drying (80 psi, 30°C, 30 minutes). After adding 100 μL of 0.1 N sodium hydroxide and 100 μL of hydrochloric acid at 30‐minute intervals, the samples were used for high‐performance liquid chromatography (HPLC)–mass spectrometry analysis.

### HPLC–Mass Spectrometry

Caffeine and its 11 downstream metabolites were separated by HPLC (Shimadzu, Kyoto, Japan) using ACQUITY UPLC BEH C18 columns (2.1 × 100 mm, 1.7 μm particle, 130 Å; Waters, Wilmslow, UK). The column temperature was set at 38°C. The HPLC system was connected to a QTRAP5500 mass spectrometer (AB Sciex, Framingham, MA). Target compounds were analyzed in a selected reaction monitoring positive ionization mode.

### Genomic DNA Analysis

At the same time as serum collection, DNA was extracted from peripheral blood according to a standard protocol using a Qiagen kit (Venlo, Netherlands). The Sanger method with an Applied Biosystems 3130 Genetic Analyzer (Life Technologies, Carlsbad, CA) was used to screen single nucleotide variations (SNVs) in genes for cytochrome P450 (CYP) 1A2 (*CYP1A2*) and cytochrome P 450 2E1 (*CYP2E1*). Pathogenicity of the identified missense variants was assessed by the sorting intolerant from tolerant method. The frequencies of each variant were evaluated using the Genome Aggregation Database (https://gnomad.broadinstitute.org).

### Statistical Analysis

Statistical analyses were performed using JMP13 (SAS Institute, Tokyo, Japan). Values under the limit of detection were replaced by β_MEAN_ calculated using the β substitution method using R version 3.6.2 (R Foundation for Statistical Computing, Vienna, Austria).^37^ The Steel‐Dwass test is a nonparametric, multiple‐comparison test and was used to examine participant characteristics and levels of caffeine and its metabolites in patients with PD, PSP, and MSA and HCs. One‐way analysis of covariance (ANCOVA) was performed using daily caffeine consumption amount as a covariate to exclude the effects of caffeine intake. Logistic regression analysis was performed to reveal the influence of sex, smoking, and alcohol. We performed ANCOVA to exclude the effects of age. Spearman's rank correlation coefficients were used to examine the relationship between serum caffeine levels and participant clinical information. *P* < 0.05 was considered statistically significant.

Analyses of minor allele frequencies among all groups were performed by chi‐square test using js‐STAR version 9.7.0j (http://www.kisnet.or.jp/nappa/software/star/freq/chisq_ixj.htm#).

### Classification of Level of Evidence

This study is rated class III because of the diagnostic case‐control study design and risk of spectrum bias.

## Results

### Participants

Participant characteristics in the first and second cohorts are shown in Tables 1 and 2, respectively. In the first cohort, patients with PSP were significantly older than patients with PD (*P* = 0.0187), whereas caffeine intake was greater in HCs compared with patients with PD (*P* = 0.0014) or PSP (*P* < 0.0001). In addition, patients with MSA had a significantly shorter disease duration (*P* = 0.0187) compared with patients with PD. Patients with PSP had a significantly lower dose of levodopa per day (*P* = 0.0285) compared with patients with PD. In the second cohort, there were no significant differences in age, sex, or daily caffeine consumption among the 4 categories. No significant differences in levodopa dose or LED were detected among groups. Patients with MSA had a shorter disease duration compared with patients with PD. Among 160 patients with PD in the validation cohort, 58 patients exhibited motor fluctuations and 26 patients experienced levodopa‐induced dyskinesia. In patients with PD, mean MDS‐UPDRS Part III scores were 13.7 ± 9.87 and 17.0 ± 13.3 points, and the mean H&Y stage was 2.09 ± 0.829 (I, 27; II, 54; III, 23; IV, 6) and 2.09 ± 0.917 (I, 42; II, 76; III, 30; IV, 9; V, 3) in the first and second cohorts, respectively. Significantly higher percentages of patients with PD, PSP, and MSA suffered from constipation compared with HCs. Lower numbers of current smokers and habitual alcohol drinkers were reported in patients with PD, PSP, and MSA compared with HCs.

**Table 2:** Participant characteristics in the second cohort

Characteristic	PD	PSP	*P* Value vs. PD	*P* Value vs. HCs	MSA	*P* Value vs. PD	*P* Value vs. HCs	HC	*P* Value vs. PD
Number	160	19			17			31	
		Probable: 15 Possible: 4			Probable: 9 Possible: 8				
Sex, male:female	63:97	8:11	0.818^b^		4:13	0.200^b^		15:16	0.350^b^
Age, y, mean (SD)	67.1 (9.17)	70.9 (7.37)	0.410^a^	0.963^a^	63.4 (10.2)	0.380^a^	0.299^a^	68.6 (11.1)	0.792^a^
Daily caffeine consumption, mg/day, mean (SD)	104 (69.0)	88.9 (89.0)	0.520^b^	0.441^b^	79.4 (69.4)	0.478^b^	0.431^b^	116 (77.1)	0.914^a^
Constipation, %	68.4	63.2	0.647^a^	0.0017^a^	82.4	0.233^a^	0.0001^a^	19.4	<0.0001^b^
(missing)	(2)	(0)			(0)			(0)	
Current smoke, %	2.50	0.00	0.486^a^	0.103^a^	0.00	0.510^a^	0.122^a^	12.9	0.0081^a^
Habitual alcohol drinking, %	24.4	10.6	0.404^a^	0.0544^a^	5.88	0.0830^a^	0.0086^a^	41.9	0.0444^a^
Disease duration, y, mean (SD)	6.39 (4.53)	4.32 (2.14)	0.180^b^		3.71 (2.08)	0.0238^b^			
LED, mg, mean (SD)	599 (318)	583 (395)	0.999^b^		449 (372)	0.200^b^			
Levodopa, mg, mean (SD)	394 (209)	511 (344)	0.229^b^		376 (282)	0.940^b^			

Caffeine intake was calculated using the Food Society Commission of Japan guidelines. Habitual alcohol drinking was defined as more than 20 g of ethanol intake more than 3 days per week according to Japanese Ministry of Health, Labour and Welfare.

a *P* value obtained by chi‐square test.

b *P* value obtained by Steel‐Dwass test.

Abbreviations: PD, Parkinson's disease; PSP, progressive supranuclear palsy; HC, heathy controls; MSA, multiple system atrophy; SD, standard deviation; LED, levodopa equivalent dose.

### Serum Levels of Caffeine and Caffeine Metabolites

In the first cohort, levels of 3,7‐dimethyluric acid, 1,3,7‐trimethyluric acid, and 1,3‐dimethyluric acid were under the limit of detection in 85.3%, 69.0%, and 96.2% of participants, respectively (data not shown). Consequently, we were unable to determine their statistical significance. As shown in Table 3, the serum caffeine level was significantly lower in patients with PD and PSP, and levels of all caffeine metabolites were significantly decreased in patients with PD, PSP, and MSA compared with HCs. We compared each metabolite under normalization of daily caffeine consumption by ANCOVA because HCs had significantly higher daily caffeine intake levels than patients with PD and PSP, but not patients with MSA (Table 4). The statistical significance of each metabolite, except for theobromine and 3‐methylxanthine, between patients with PD or PSP and HCs under normalized conditions of caffeine intake was confirmed (caffeine, *F* = 24.8, *P* < 0.0001 in PD vs. HC and *F* = 14.4, *P* = 0.0003 in PSP vs. HC). Although significantly decreased levels of all metabolites were identified in patients with PSP compared with patients with PD (Table 3), only levels of theophylline (*F* = 4.92, *P* = 0.0282), theobromine (*F* = 4.20, *P* = 0.0424), 7‐methylxanthine (*F* = 6.27, *P* = 0.0135), and AAMU (*F* = 4.83, *P* = 0.0296) were significantly decreased in patients with PSP with normalized with caffeine intake, implying limited practical utility for differential diagnosis (Table 4). Although caffeine metabolic profiles were uniformly decreased in patients with PD, PSP, and MSA compared with HCs, this decreased tendency was particularly evident in patients with PSP.

**Table 3:** Alterations in levels of caffeine and its downstream metabolites in patients with PD, PSP, and MSA and HCs in the first cohort

	PD			PSP				MSA				HC	
Compound Name	Mean ± SE, μmol/L	LLD	*P* Value vs. HCs	Mean ± SE, μmol/L	LLD	*P* Value vs. PD	*P* Value vs. HCs	Mean ± SE, μmol/L	LLD	*P* Value vs. PD	*P* Value vs. HCs	Mean ± SE, μmol/L	LLD
Caffeine	2.45 ± 0.241	5	<0.0001	1.49 ± 0.334	8	0.0388	<0.0001	3.81 ± 1.16	3	0.988	0.0670	5.60 ± 0.661	1
Theophylline	0.539 ± 0.0425	4	<0.0001	0.291 ± 0.0558	6	0.0094	<0.0001	0.527 ± 0.115	2	0.869	0.0040	1.04 ± 0.103	1
Theobromine	2.27 ± 0.241	1	0.0024	1.21 ± 0.255	4	0.0169	<0.0001	1.61 ± 0.313	2	0.566	0.0101	3.40 ± 0.453	1
Paraxanthine	2.43 ± 0.182	2	<0.0001	1.51 ± 0.318	5	0.0171	<0.0001	2.58 ± 0.588	2	0.932	0.0036	5.54 ± 0.618	1
1,7‐Dimethyluric acid	0.0716 ± 0.00660	11	<0.0001	0.0461 ± 0.0112	13	0.0087	<0.0001	0.0854 ± 0.0193	7	0.995	0.0049	0.180 ± 0.0238	1
1‐Methylxanthine	0.0715 ± 0.00609	9	<0.0001	0.0418 ± 0.00805	9	0.0096	<0.0001	0.0615 ± 0.0107	6	0.888	0.0009	0.152 ± 0.0195	1
3‐Methylxanthine	0.164 ± 0.0200	7	0.0029	0.0833 ± 0.0200	10	0.0026	<0.0001	0.102 ± 0.0195	3	0.396	0.0008	0.274 ± 0.0478	1
7‐Methylxanthine	0.172 ± 0.0148	4	0.0008	0.0880 ± 0.0207	9	0.0013	<0.0001	0.0952 ± 0.0160	3	0.0957	<0.0001	0.295 ± 0.0433	1
AAMU	0.526 ± 0.0410	9	<0.0001	0.290 ± 0.0654	10	0.0023	<0.0001	0.374 ± 0.0705	5	0.291	<0.0001	1.10 ± 0.109	1

*P* values versus PD are shown first, followed by those versus HC. Values below the LLD were calculated using the β‐substitution method. Each number in the LLD cells shows each sample number below LLD. *P* value obtained by Steel‐Dwass test.

Abbreviations: PD, Parkinson's disease; PSP, progressive supranuclear palsy; MSA, multiple system atrophy; HC, healthy controls; SE, standard error; LLD, lower limits of detection; AAMU, 5‐acethylamino‐6‐amino‐3‐methyluracil.

**Table 4:** Comparison of *F* values and *P* values in each group of the first cohort under normalization of daily caffeine consumption amount

Compound Name	PD vs. PSP		PD vs. MSA		PD vs. HCs		PSP vs. HCs		MSA vs. HCs	
	*F* Value	*P* Value	*F* Value	*P* Value	*F* Value	*P* Value	*F* Value	*P* Value	*F* Value	*P* Value
Caffeine	1.99	0.160	3.35	0.0695	24.8	<0.0001	14.4	0.0003	2.10	0.152
Theophylline	4.92	0.0282	0.0199	0.888	20.8	<0.0001	18.6	<0.0001	7.95	0.0064
Theobromine	4.20	0.0424	1.41	0.238	4.17	0.0429	7.59	0.0075	5.22	0.0257
Paraxanthine	3.37	0.0686	0.0789	0.779	33.3	<0.0001	15.4	0.0002	8.32	0.0054
1,7‐Dimethyluric acid	1.40	0.239	0.675	0.413	26.1	<0.0001	9.28	0.0033	4.67	0.0345
1‐Methylxanthine	3.68	0.0573	0.533	0.467	20.8	<0.0001	12.4	0.0008	8.48	0.005
3‐Methylxanthine	3.79	0.0535	2.83	0.0950	3.75	0.0546	4.01	0.0492	3.97	0.0506
7‐Methylxanthine	6.27	0.0135	5.45	0.0210	7.56	0.0067	6.25	0.0147	7.20	0.0093
AAMU	4.83	0.0296	2.71	0.102	25.8	<0.0001	15.04	0.0002	15.3	0.0002

*F* values and *P* values obtained by analysis of covariance.

Abbreviations: PD, Parkinson's disease; PSP, progressive supranuclear palsy; MSA, multiple system atrophy; HC, healthy controls; AAMU, 5‐acethylamino‐6‐amino‐3‐methyluracil.

In the second cohort, 3,7‐dimethyluric acid and 1,3,7‐trimethyluric acid levels were under the limit of detection in 90.9% and 73.5% of participants, respectively, and could not be assessed statistically (data not shown). Levels of caffeine and 5 downstream metabolites (theophylline, paraxanthine, 1,3‐trimethyl uric acid, 1,7‐dimethyluric acid, and AAMU) were significantly lower in patients with PD, PSP, and MSA compared with HCs (Table 5). Levels of 1‐methylxanthine were significantly decreased in patients with PD and MSA only. No significantly decreased levels of 3‐methylxanthine and 7‐methylxanthine were detected in any disease. All examined metabolites that tended to be decreased in the 3 parkinsonian disorders compared with HCs were confirmed in the second cohort. However, this decreased tendency was smaller in patients with PSP, which was not consistent with the results of the first cohort. In addition, in the second cohort, patients with PD, PSP, or MSA tended to take less caffeine than HCs without significant differences (Table 2). Similar to the analysis of the first cohort, ANCOVA using daily caffeine intake as a covariate revealed decreased levels of all 10 metabolites in the 3 parkinsonian disorders (Supplementary Table 1). When combined with results of the first cohort (Table 4), of 9 metabolites detected in more than 50% of the participants in both cohorts, 8 in PD, 5 in PSP, and 3 in MSA were significantly reduced compared with HCs.

**Table 5:** Alterations in caffeine and its downstream metabolite levels in patients with PD, PSP, and MSA and HCs in the second cohort

	PD			PSP				MSA				HCs	
Compound Name	Mean ± SE, μmol/L	LLD	*P* Value vs. HCs	Mean ± SE, μmol/L	LLD	*P* Value vs. PD	*P* Value vs. HCs	Mean ± SE, μmol/L	LLD	*P* Value vs. PD	*P* Value vs. HCs	Mean ± SE, μmol/L	LLD
Caffeine	2.95 ± 0.295	5	<0.0001	4.33 ± 1.55	1	0.988	0.0393	2.10 ± 0.630	4	0.587	0.0021	8.75 ± 1.57	0
Theophylline	0.557 ± 0.0369	2	<0.0001	0.613 ± 0.133	1	1.00	0.0089	0.729 ± 0.364	3	0.688	0.003	1.25 ± 0.162	0
Theobromine	2.37 ± 0.180	0	0.0201	3.37 ± 0.912	0	0.998	0.463	1.99 ± 0.546	2	0.672	0.115	5.08 ± 1.56	0
Paraxanthine	2.60 ± 0.170	2	<0.0001	3.67 ± 0.770	0	0.673	0.0438	2.40 ± 0.596	3	0.882	0.002	7.07 ± 1.09	0
1,3‐Dimethyluric acid	0.0300 ± 0.00162	28	<0.0001	0.0373 ± 0.00710	3	0.958	0.0174	0.0493 ± 0.0274	6	0.401	0.0013	0.0747 ± 0.0104	0
1,7‐Dimethyluric acid	0.0854 ± 0.00578	10	<0.0001	0.103 ± 0.0206	1	0.929	0.0242	0.0685 ± 0.0178	6	0.508	0.0009	0.215 ± 0.0299	0
1‐Methylxanthine	0.0933 ± 0.00597	6	<0.0001	0.150 ± 0.0372	1	0.957	0.174	0.104 ± 0.0407	5	0.622	0.0074	0.230 ± 0.0379	0
3‐Methylxanthine	0.177 ± 0.0128	4	0.148	0.214 ± 0.0617	0	0.978	0.463	0.178 ± 0.0564	5	0.505	0.229	0.311 ± 0.0992	0
7‐Methylxanthine	0.212 ± 0.0148	3	0.0778	0.319 ± 0.0848	0	1.00	0.732	0.203 ± 0.0561	4	0.847	0.268	0.377 ± 0.0908	0
AAMU	0.592 ± 0.0369	3	0.0004	0.627 ± 0.144	1	0.992	0.0297	0.524 ± 0.124	4	0.763	0.0126	1.19 ± 0.155	0

*P* values versus PD are shown first, followed by those versus HC. Values below the LLD were calculated using the β‐substitution method. Each number in the LLD cells shows each sample number below LLD. *P* value obtained by Steel‐Dwass test.

Abbreviations: PD, Parkinson's disease; PSP, progressive supranuclear palsy; MSA, multiple system atrophy; HC, healthy controls; SE, standard error; LLD, lower limits of detection; AAMU, 5‐acethylamino‐6‐amino‐3‐methyluracil.

### Association of Caffeine Metabolites With Clinical Parameters and Medication

Because patients with PD, PSP, and MSA commonly suffered from significantly more episodes of constipation compared with HCs, we examined the relationship between clinical parameters (disease severity, disease duration, constipation) and levels of serum caffeine and its metabolites in the second cohort (Supplementary Table 2). In PD, caffeine levels showed a slight negative correlation with H&Y (Spearman's rank correlation coefficient, ρ = −0.215, *P* = 0.0063) and MDS‐UPDRS Part III (ρ = −0.250, *P* = 0.0015). Correlation coefficients of other downstream metabolites of caffeine with H&Y or MDS‐UPDRS Part III ranged from −0.294 to 0.0076, suggesting similar slight correlations, consistent with our previous study.^18^ Disease duration also showed a significant but weak correlation with caffeine (ρ = −0.272, *P* = 0.0005) and its downstream metabolite levels. There were no significant differences in caffeine levels between patients with PD with or without constipation (*P* = 0.681). No notable differences in downstream metabolites were detected (Supplementary Table 3).

We also examined the correlation between antiparkinsonian drugs and caffeine in the second cohort because all patients with PD were under dopaminergic treatment (Supplementary Table 4). Accordingly, both LED and levodopa in PD showed a significant inverse correlation with serum levels of caffeine (LED, ρ = −0.313, *P* < 0.0001; levodopa, ρ = −0.244, *P* = 0.0019) and some of its metabolites. There were no significant correlations between LED or levodopa dose in patients with PSP or MSA and each metabolite.

### Association of Caffeine Metabolites With Age and Sex

Because of the significant differences in age among patients with PD and PSP and HCs in the first cohort (Table 1), we performed an ANCOVA between each pair of groups using age as a covariate. Significant differences between the 3 disease groups and HCs did not change between before and after normalization (Table 3 and Supplementary Table 5, respectively). An ANCOVA performed in the second cohort showed similar results (Table 5, Supplementary Table 6). Levels of caffeine between patients with PSP and HCs (*P* = 0.0568) and those of theophylline and 1,3‐dimethyluric acid between patients with MSA and HCs (*P* = 0.0681 and 0.180, respectively) were not significant.

Sex ratios in both cohorts were not fully matched and were without significant differences. Correlations of caffeine intake with each metabolite concentration were not significantly different between sexes (Supplementary Table 7), revealing sex has no significant effect on the association between caffeine intake and each analyte level.

### Association of Caffeine Metabolites With Smoking and Habitual Alcohol Drinking

The half‐life of caffeine is shortened by smoking and prolonged by alcohol.^35^ We used logistic regression models to reveal the effects on the association between caffeine intake and each analyte in PD patients in the second cohort (Supplementary Table 8). Smoking or alcohol had no significant effect on the association between daily caffeine intake and each analyte level. We could not perform the same analysis in patients with PSP and MSA because of sample size limitations.

### Analysis of Genes Associated With Caffeine Metabolism

Based on the publicly available Genome Aggregation Database and our previous study, we identified 5 *CYP1A2* SNVs and 3 *CYP2E1* SNVs associated with caffeine metabolism.^18^ There were no significant differences in the frequencies of any SNV among patients with PD, PSP, and MSA or HCs (Supplementary Table 9).

## Discussion

In the current study, we identified significantly decreased levels of caffeine in patients with PD, PSP, and MSA in both cohorts. Four downstream metabolites (theophylline, paraxanthine, 1,7‐dimethyluric acid, and AAMU) were significantly suppressed in all 3 diseases in both cohorts. Even in the first cohort with an unequal age distribution across the 3 diseases, 6 downstream metabolites (theophylline, paraxanthine, 1,7‐dimethyluric acid, 1‐methylxanthine, and 7‐methylxanthine, and AAMU) were consistently decreased in all parkinsonian disorders. Even after normalizing to daily caffeine consumption, the decreased tendency in all parkinsonian disorders was preserved, and 8 of 9 metabolites in patients with PD, 5 of 9 in patients with PSP, and 3 of 9 in patients with MSA were significantly decreased compared with HCs.

Compared with our previous report analyzing caffeine and its 11 downstream metabolites, the current study excluded 5‐acethylamino‐6‐formylamino‐3‐methyluracil measurements because it is unstable related to its spontaneous conversion to AAMU in the absence of enzymic activity.^38^ In our previous report, caffeine and its 9 downstream metabolites (theophylline, theobromine, paraxanthine, 1,7‐dimethyluric acid, 1‐methylxanthine, 3‐methylxanthine, 7‐methylxanthine, 5‐acethylamino‐6‐formylamino‐3‐methyluracil, and AAMU) were significantly decreased in patients with PD compared with HCs. As shown in Tables 3, 4, 5 and Supplementary Table 1, we confirmed a similar caffeine metabolic profile in patients with PD of both cohorts, indicating the high reliability of this double cohort study based on the reproducibility of our previous study.^18^

Theobromine levels were consistently decreased in all 3 diseases, but in patients with PD (first cohort), patients with PSP (second cohort), and patients with MSA (second cohort) they were not significant. Levels of theobromine, a principle alkaloid contained in various foods including *Theobroma cacao* (https://pubchem.ncbi.nlm.nih.gov/compound/5429), might be affected by internal caffeine metabolism and exogenous intake because dietary intake of theobromine was not matched among the groups.

Although patients with PD or MSA were reported to drink lower amounts of coffee,^15^, ^39^, ^40^, ^41^ this is controversial.^16^, ^42^ In the first cohort, caffeine consumption in patients with PD and PSP was lower compared with HCs, and a similar nonsignificant tendency was detected in patients with MSA. However, we confirmed decreased levels of caffeine and its metabolites in the 3 diseases under conditions normalized by daily caffeine consumption in the first cohort. Although similar results under conditions normalized by daily caffeine consumption in the second cohort were reported, especially in patients with PD, the statistical significance was lower in patients with PSP or MSA compared with HCs, possibly because of the limited sample numbers of patients with PSP and MSA in the second cohort (*n* = 19 and 17, respectively).

Similarly decreased levels of caffeine and its downstream metabolites in the 3 diseases suggests a common disease pathway that might involve caffeine malabsorption, its hypermetabolism, and/or increased renal clearance. In the current study, mild but significant correlations between levodopa or LED and each absolute concentration of caffeine and its metabolites were detected in both cohorts (Supplementary Table 4). Caffeine is passively absorbed from the lumen into the small intestinal mucosa.^43^ Levodopa is also absorbed from the small intestine by transporters including amino acid transporter‐related to b0,+ amino acid transporter (b0,+ AT‐rBAT), L‐amino acid transporter‐4F2 heavy chain (LAT2‐4F2hc), and T‐type amino acid transporter (TAT1),^44^ without reported evidence of a direct molecular interaction between caffeine and levodopa and competitive binding of caffeine with the transporters. Considering the common profile of caffeine metabolites among the 3 diseases, we cannot exclude the possibility that levodopa might be absorbed competitively with caffeine. In terms of hypermetabolism of caffeine and its metabolites, haptic CYP1A2, CYP3A4, or CYP3A5 catabolize levodopa and caffeine.^38^, ^45^ Caffeine metabolism is influenced by many drugs, especially those affecting the activity of CYP1A2 via autoinduction.^46^ Thus, the upregulated expression of CYPs by administration of levodopa or other antiparkinsonian drugs in the 3 diseases might lead to the collateral hypermetabolism of caffeine.

The gut–brain axis might be a potential mechanism related to PD that affects infection, neuroinflammation, and the spread of alpha‐synuclein.^47^ Gut microbiome profiles are changed in patients with PD compared with HCs.^48^, ^49^, ^50^ Likewise, the profiles of patients with PSP and MSA are similar to those of patients with PD.^50^, ^51^ Although the small intestinal microbiome fluctuated more easily in response to the latest diet trends compared with the gut microbiome,^52^ the small intestinal microbiome may have a common pathogenic tendency that affects chemical absorption in the 3 diseases.^53^

Caffeine metabolism or renal excretion of caffeine might be increased in the 3 diseases. Oral caffeine is absorbed completely and metabolized exclusively in the liver, and metabolites are excreted in urine, with <3% of caffeine unmetabolized. Fasting caffeine concentration reflects caffeine clearance via the liver.^33^, ^54^ Despite no significant differences in the frequency of SNVs associated with caffeine metabolism, epigenetic and/or environmental alteration might affect hepatic or renal functions in patients with parkinsonian disorders.

Our results suggest a possible reason why oral caffeine intake in the Café‐PD study was not beneficial for the motor symptoms of patients with PD despite evidence for caffeine efficacy against motor symptoms.^26^ Caffeine might have a better outcome if other routes of administration, such as transdermal, sublingual, intravenous, or transrectal, are used.

The study had some limitations. First, it was conducted at a single university hospital and severe cases of PD (H&Y IV and V) were not fully represented because of our strict exclusion criteria. PD, PSP, and MSA were diagnosed clinically without pre/postsynaptic imaging or pathological confirmation with the inclusion of possible PSP and possible MSA (Tables 1 and 2). The number of patients with PSP or MSA were limited. Although there were differences in caffeine intake and age at sampling among patients with PD and PSP and HCs in both cohorts, decreased levels of caffeine and its downstream metabolites were confirmed under their normalized conditions. We also confirmed sex, smoking, and alcohol had no significant effects on levels of each metabolite. We could not exclude the possibility that exogenous chemicals contained in coffee or green tea might have affected caffeine metabolism or excretion because of technical limitations of the measurement system using liquid chromatography–mass spectrometry.

We confirmed a uniform decrease of caffeine and its downstream metabolites in PD and identified their consistent decrease in PSP and MSA. Our data suggest that this set of metabolites would not be useful for the differential diagnosis of these diseases. However, a common mechanism such as malabsorption or increased metabolism/clearance of caffeine may underlie the 3 parkinsonian diseases.

## Author Roles

(1) Research Project: A. Conception and Design, B. Acquisition of Data, C. Organization; (2) Statistical Analysis: A. Design, B. Execution, C. Review and Critique; (3) Manuscript: A. Writing of the first draft, B Review and Critique; (4) Others: A. Technical and Material Supports, B. Management of Data, C. Study Supervision, D. Supervision of Data Collection.

H.T.: 1A, 1B, 2A, 2B, 3A

S.S.: 1A, 1B, 1C, 2A, 2B, 2C, 3A, 3B

M.F.: 1A, 1B, 3B

S.‐I.U.: 1B, 3B

Y.L.: 4A, 3B

T.H.: 1A, 1B, 3B

K.‐I.I.: 1B, 3B

Y.O.: 1B, 3B

A.M.: 1B, 3B

A.O.: 1B, 3B

T.T.: 1B, 3B

K.D.: 1B, 3B

Y. Ishiguro: 1B, 3B

Y. Imamichi: 4A, 4B

H.N.: 2B, 2C, 4A

S.N.: 2B, 2C, 4A

M.F.: 3B, 4A

N.H.: 1A, 1C, 2A, 2C, 3B, 4C, 4D

## Financial Disclosures of all authors (for the preceding 12 months)

N.H. received personal fees from Hisamitsu Pharmaceutical, Dai‐Nippon Sumitomo Pharma, Otsuka Pharmaceutical, Novartis Pharma, GlaxoSmithKline, Nippon Boehringer Ingelheim, FP Pharmaceutical, Eisai, Kissei Pharmaceutical, Janssen Pharmaceutical, Nihon Medi‐Physics, and Kyowa Hakko‐Kirin (outside of this study). The other authors have nothing to report.

## Supporting information


**Supplementary Table 1** Comparison of *F*‐values and *p*‐values in each group of the 2^nd^ cohort under normalization of daily caffeine consumption amount.
Supplementary Table 2. Correlation between caffeine metabolites and disease severity and disease duration in Parkinson's disease patients in the 2^nd^ cohort.
Supplementary Table 3. Association between caffeine metabolites and constipation in Parkinson's disease patients in the 2^nd^ cohort.
Supplementary Table 4. Correlation between caffeine metabolites and anti‐parkinsonian drugs in the 2^nd^ cohort.
Supplementary Table 5. Comparison of *F*‐values and *p*‐values in each group of the 1^st^ cohort under normalization of age.
Supplementary Table 6. Comparison of *F*‐values and *p*‐values in each group of the 2^nd^ cohort under normalization of age.
Supplementary Table 7. Correlations of caffeine intake with each metabolite concentration were not significantly different between genders in Parkinson's disease patients.
Supplementary Table 8. Correlations between daily caffeine intake and each analyte level were not affected by smoking or alcohol habit in Parkinson's disease patients in the 2^nd^ cohort.
Supplementary Table 9. Alternative minor allele frequencies of *CYP1A2* and *CYP2E1* variants in both cohorts.

**Supplementary Figure**. Metabolic pathways of caffeine and its metabolites measured in the current study

## References

[1] Jankovic J , Poewe W . Therapies in Parkinson's disease. Curr Opin Neurol 2012;25(4):433–447. 22691758 10.1097/WCO.0b013e3283542fc2

[2] Postuma RB , Berg D , Stern M , et al. MDS clinical diagnostic criteria for Parkinson's disease. Mov Disord 2015;30(12):1591–1601. 26474316 10.1002/mds.26424

[3] Schapira AHV , Chaudhuri KR , Jenner P . Non‐motor features of Parkinson disease. Nat Rev Neurosci 2017;18(8):509. 28720825 10.1038/nrn.2017.91

[4] Papp MI , Kahn JE , Lantos PL . Glial cytoplasmic inclusions in the CNS of patients with multiple system atrophy (striatonigral degeneration, olivopontocerebellar atrophy and Shy‐Drager syndrome). J Neurol Sci 1989;94(1‐3):79–100. 2559165 10.1016/0022-510x(89)90219-0

[5] Nakazato Y , Yamazaki H , Hirato J , Ishida Y , Yamaguchi H . Oligodendroglial microtubular tangles in olivopontocerebellar atrophy. J Neuropath Exp Neur 1990;49(5):521–530. 1703225 10.1097/00005072-199009000-00007

[6] Hauw JJ , Daniel SE , Dickson D , et al. Preliminary NINDS neuropathologic criteria for Steele‐Richardson‐Olszewski syndrome (progressive supranuclear palsy). Neurology 1994;44(11):2015–2019. 7969952 10.1212/wnl.44.11.2015

[7] Litvan I , Hauw JJ , Bartko JJ , et al. Validity and reliability of the preliminary NINDS neuropathologic criteria for progressive supranuclear palsy and related disorders. J Neuropath Exp Neur 1996;55(1):97–105. 8558176 10.1097/00005072-199601000-00010

[8] Kolahdouzan M , Hamadeh MJ . The neuroprotective effects of caffeine in neurodegenerative diseases. CNS Neurosci Ther 2017;23(4):272–290. 28317317 10.1111/cns.12684 PMC6492672

[9] Nishitsuji K , Watanabe S , Xiao J , et al. Effect of coffee or coffee components on gut microbiome and short‐chain fatty acids in a mouse model of metabolic syndrome. Sci Rep 2018;8(1):16173. 30385796 10.1038/s41598-018-34571-9 PMC6212590

[10] Scheperjans F , Pekkonen E , Kaakkola S , Auvinen P . Linking smoking, coffee, urate, and Parkinson's disease—a role for gut microbiota? J Parkinsons Dis 2015;5(2):255–262. 25882059 10.3233/JPD-150557

[11] Derkinderen P , Shannon KM , Brundin P . Gut feelings about smoking and coffee in Parkinson's disease. Mov Disord 2014;29(8):976–979. 24753353 10.1002/mds.25882 PMC4107006

[12] Chen X , Ghribi O , Geiger JD . Caffeine protects against disruptions of the blood‐brain barrier in animal models of Alzheimer's and Parkinson's diseases. J Alzheimers Dis 2010;20(suppl 1):S127–S141. 20164568 10.3233/JAD-2010-1376 PMC3086010

[13] Calon F , Dridi M , Hornykiewicz O , Bedard PJ , Rajput AH , Di Paolo T . Increased adenosine A2A receptors in the brain of Parkinson's disease patients with dyskinesias. Brain 2004;127(Pt 5):1075–1084. 15033896 10.1093/brain/awh128

[14] Popat RA , Van Den Eeden SK , Tanner CM , et al. Coffee, ADORA2A, and CYP1A2: the caffeine connection in Parkinson's disease. Eur J Neurol 2011;18(5):756–765. 21281405 10.1111/j.1468-1331.2011.03353.x PMC3556904

[15] Tan EK , Lu ZY , Fook‐Chong SM , et al. Exploring an interaction of adenosine A2A receptor variability with coffee and tea intake in Parkinson's disease. Am J Med Genet B Neuropsychiatr Genet 2006;141B(6):634–636. 16823803 10.1002/ajmg.b.30359

[16] Facheris MF , Schneider NK , Lesnick TG , et al. Coffee, caffeine‐related genes, and Parkinson's disease: a case‐control study. Mov Disord 2008;23(14):2033–2040. 18759349 10.1002/mds.22247 PMC4554698

[17] Chuang YH , Lill CM , Lee PC , et al. Gene‐environment interaction in Parkinson's disease: coffee, ADORA2A, and CYP1A2. Neuroepidemiology 2016;47(3–4):192–200. 28135712 10.1159/000450855 PMC5465963

[18] Fujimaki M , Saiki S , Li Y , et al. Serum caffeine and metabolites are reliable biomarkers of early Parkinson disease. Neurology 2018;90(5):e404–e411. 29298852 10.1212/WNL.0000000000004888 PMC5791797

[19] Ross GW , Abbott RD , Petrovitch H , et al. Association of coffee and caffeine intake with the risk of Parkinson disease. JAMA 2000;283(20):2674–2679. 10819950 10.1001/jama.283.20.2674

[20] Liu R , Guo X , Park Y , et al. Caffeine intake, smoking, and risk of Parkinson disease in men and women. Am J Epidemiol 2012;175(11):1200–1207. 22505763 10.1093/aje/kwr451 PMC3370885

[21] Hernan MA , Takkouche B , Caamano‐Isorna F , Gestal‐Otero JJ . A meta‐analysis of coffee drinking, cigarette smoking, and the risk of Parkinson's disease. Ann Neurol 2002;52(3):276–284. 12205639 10.1002/ana.10277

[22] Ascherio A , Zhang SM , Hernan MA , et al. Prospective study of caffeine consumption and risk of Parkinson's disease in men and women. Ann Neurol 2001;50(1):56–63. 11456310 10.1002/ana.1052

[23] Ascherio A , Schwarzschild MA . The epidemiology of Parkinson's disease: risk factors and prevention. Lancet Neurol 2016;15(12):1257–1272. 27751556 10.1016/S1474-4422(16)30230-7

[24] Moccia M , Erro R , Picillo M , et al. Caffeine consumption and the 4‐year progression of de novo Parkinson's disease. Parkinsonism Relat Disord 2016;32:116–119. 27622969 10.1016/j.parkreldis.2016.08.005

[25] Postuma RB , Lang AE , Munhoz RP , et al. Caffeine for treatment of Parkinson disease: a randomized controlled trial. Neurology 2012;79(7):651–658. 22855866 10.1212/WNL.0b013e318263570d PMC3414662

[26] Postuma RB , Anang J , Pelletier A , et al. Caffeine as symptomatic treatment for Parkinson disease (Cafe‐PD): a randomized trial. Neurology 2017;89(17):1795–1803. 28954882 10.1212/WNL.0000000000004568 PMC5664303

[27] Hatano T , Saiki S , Okuzumi A , Mohney RP , Hattori N . Identification of novel biomarkers for Parkinson's disease by metabolomic technologies. J Neurol Neurosurg Psychiatry 2016;87(3):295–301. 25795009 10.1136/jnnp-2014-309676

[28] Han W , Sapkota S , Camicioli R , Dixon RA , Li L . Profiling novel metabolic biomarkers for Parkinson's disease using in‐depth metabolomic analysis. Mov Disord 2017;32(12):1720–1728. 28880465 10.1002/mds.27173 PMC5753769

[29] Hoglinger GU , Respondek G , Stamelou M , et al. Clinical diagnosis of progressive supranuclear palsy: the Movement Disorder Society criteria. Mov Disord 2017;32(6):853–864. 28467028 10.1002/mds.26987 PMC5516529

[30] Gilman S , Wenning GK , Low PA , et al. Second consensus statement on the diagnosis of multiple system atrophy. Neurology 2008;71(9):670–676. 18725592 10.1212/01.wnl.0000324625.00404.15 PMC2676993

[31] Goetz CG , Tilley BC , Shaftman SR , et al. Movement Disorder Society‐sponsored revision of the Unified Parkinson's Disease Rating Scale (MDS‐UPDRS): scale presentation and clinimetric testing results. Mov Disord 2008;23(15):2129–2170. 19025984 10.1002/mds.22340

[32] Tomlinson CL , Stowe R , Patel S , Rick C , Gray R , Clarke CE . Systematic review of levodopa dose equivalency reporting in Parkinson's disease. Mov Disord 2010;25(15):2649–2653. 21069833 10.1002/mds.23429

[33] Lelo A , Miners JO , Robson R , Birkett DJ . Assessment of caffeine exposure: caffeine content of beverages, caffeine intake, and plasma concentrations of methylxanthines. Clin Pharmacol Ther 1986;39(1):54–59. 3943270 10.1038/clpt.1986.10

[34] Moon C‐M , Jeong G‐W . Associations of neurofunctional, morphometric and metabolic abnormalities with clinical symptom severity and recognition deficit in obsessive–compulsive disorder. J Affect Disord 2018;227:603–612. 29172053 10.1016/j.jad.2017.11.059

[35] Preedy VR, ed. Caffeine: Chemistry, Analysis, Function and Effects. London, UK: Royal Society of Chemistry; 2012.

[36] Che B , Wang L , Zhang Z , Zhang Y , Deng Y . Distribution and accumulation of caffeine in rat tissues and its inhibition on semicarbazide‐sensitive amine oxidase. Neurotoxicology 2012;33(5):1248–1253. 22841599 10.1016/j.neuro.2012.07.004

[37] Ganser GH , Hewett P . An accurate substitution method for analyzing censored data. J Occup Environ Hyg 2010;7(4):233–244. 20169489 10.1080/15459621003609713

[38] Rybak ME , Pao CI , Pfeiffer CM . Determination of urine caffeine and its metabolites by use of high‐performance liquid chromatography‐tandem mass spectrometry: estimating dietary caffeine exposure and metabolic phenotyping in population studies. Anal Bioanal Chem 2014;406(3):771–784. 24306330 10.1007/s00216-013-7506-9

[39] Fall PA , Fredrikson M , Axelson O , Granerus AK . Nutritional and occupational factors influencing the risk of Parkinson's disease: a case‐control study in southeastern Sweden. Mov Disord 1999;14(1):28–37. 9918341 10.1002/1531-8257(199901)14:1<28::aid-mds1007>3.0.co;2-o

[40] Preux PM , Condet A , Anglade C , et al. Parkinson's disease and environmental factors. Matched case‐control study in the Limousin region, France. Neuroepidemiology 2000;19(6):333–337. 11060508 10.1159/000026273

[41] Seo JH , Yong SW , Song SK , Lee JE , Sohn YH , Lee PH . A case‐control study of multiple system atrophy in Korean patients. Mov Disord 2010;25(12):1953–1959. 20623770 10.1002/mds.23185

[42] Morano A , Jimenez‐Jimenez FJ , Molina JA , Antolin MA . Risk‐factors for Parkinson's disease: case‐control study in the province of Caceres, Spain. Acta Neurol Scand 1994;89(3):164–170. 8030397 10.1111/j.1600-0404.1994.tb01655.x

[43] Kalow W , Tang BK . The use of caffeine for enzyme assays: a critical appraisal. Clin Pharmacol Ther 1993;53(5):503–514. 8491061 10.1038/clpt.1993.63

[44] Camargo SM , Vuille‐dit‐Bille RN , Mariotta L , et al. The molecular mechanism of intestinal levodopa absorption and its possible implications for the treatment of Parkinson's disease. J Pharmacol Exp Ther 2014;351(1):114–123. 25073474 10.1124/jpet.114.216317

[45] Cacabelos R. Parkinson's disease: from pathogenesis to pharmacogenomics. Int J Mol Sci 2017;18(3). 10.3390/ijms18030551 PMC5372567 28273839

[46] Nehlig A. Interindividual differences in caffeine metabolism and factors driving caffeine consumption. Pharmacol Rev 2018;70(2):384–411. 29514871 10.1124/pr.117.014407

[47] Breen DP , Halliday GM , Lang AE . Gut‐brain axis and the spread of alpha‐synuclein pathology: vagal highway or dead end? Mov Disord 2019;34(3):307–316. 30653258 10.1002/mds.27556

[48] Keshavarzian A , Green SJ , Engen PA , et al. Colonic bacterial composition in Parkinson's disease. Mov Disord 2015;30(10):1351–1360. 26179554 10.1002/mds.26307

[49] Scheperjans F , Aho V , Pereira PA , et al. Gut microbiota are related to Parkinson's disease and clinical phenotype. Mov Disord 2015;30(3):350–358. 25476529 10.1002/mds.26069

[50] Gerhardt S , Mohajeri MH . Changes of colonic bacterial composition in Parkinson's disease and other neurodegenerative diseases. Nutrients 2018;10(6): pii:E708 10.3390/nu10060708 29857583 PMC6024871

[51] Barichella M , Severgnini M , Cilia R , et al. Unraveling gut microbiota in Parkinson's disease and atypical parkinsonism. Mov Disord 2019;34(3):396–405. 30576008 10.1002/mds.27581

[52] El Aidy S , van den Bogert B , Kleerebezem M . The small intestine microbiota, nutritional modulation and relevance for health. Curr Opin Biotechnol 2015;32:14–20. 25308830 10.1016/j.copbio.2014.09.005

[53] Fasano A , Bove F , Gabrielli M , et al. The role of small intestinal bacterial overgrowth in Parkinson's disease. Mov Disord 2013;28(9):1241–1249. 23712625 10.1002/mds.25522

[54] Renner E , Wietholtz H , Huguenin P , Arnaud MJ , Preisig R . Caffeine: a model compound for measuring liver function. Hepatology 1984;4(1):38–46. 6420303 10.1002/hep.1840040107

