# Supplementary material for: Shared Metabolic Profile of Caffeine in Parkinsonian Disorders
Source: Mov Disord. 2020 May 1;35(8):1438–47. doi: 10.1002/mds.28068 (PMC7496239; doi:10.1002/mds.28068)
Supplement: Supplementary file 1 — Supplementary Table 1 Comparison of F‐values and p‐values in each group of the 2nd cohort under normalization of daily caffeine consumption amount. Supplementary Table 2. Correlation between caffeine metabolites and disease severity and disease duration in Parkinson's disease patients in the 2nd cohort. Supplementary Table 3. Association between caffeine metabolites and constipation in Parkinson's disease patients in the 2nd cohort. Supplementary Table 4. Correlation between caffeine metabolites and anti‐parkinsonian drugs in the 2nd cohort. Supplementary Table 5. Comparison of F‐values and p‐values in each group of the 1st cohort under normalization of age. Supplementary Table 6. Comparison of F‐values and p‐values in each group of the 2nd cohort under normalization of age. Supplementary Table 7. Correlations of caffeine intake with each metabolite concentration were not significantly different between genders in Parkinson's disease patients. Supplementary Table 8. Correlations between daily caffeine intake and each analyte level were not affected by smoking or alcohol habit in Parkinson's disease patients in the 2nd cohort. Supplementary Table 9. Alternative minor allele frequencies of CYP1A2 and CYP2E1 variants in both cohorts. [file MDS-35-1438-s001.docx]

**Supplementary Table 1. Comparison of *F*-values and *p*-values in each group of the 2^nd^ cohort under normalization of daily caffeine consumption amount.**

|  | PD vs PSP | | PD vs MSA | | PD vs HC |  | PSP vs HC | | MSA vs HC | |
| --- | --- | --- | --- | --- | --- | --- | --- | --- | --- | --- |
|  | *F*-value | *p*-value | *F*-value | *p*-value | *F*-value | *p*-value | *F*-value | *p*-value | *F*-value | *p*-value |
| caffeine | 2.70 | 0.102 | 0.238 | 0.626 | 35.4 | <.0001 | 3.07 | 0.0863 | 8.68 | 0.0051 |
| theophylline | 0.772 | 0.381 | 3.14 | 0.0784 | 38.7 | <.0001 | 6.67 | 0.0130 | 1.45 | 0.2349 |
| theobromine | 3.07 | 0.0814 | 0.0676 | 0.795 | 11.4 | 0.0009 | 0.603 | 0.442 | 1.71 | 0.1982 |
| paraxanthine | 6.18 | 0.0138 | 0.142 | 0.707 | 52.5 | <.0001 | 4.10 | 0.0485 | 7.42 | 0.0092 |
| 1,3-dimethyluric acid | 3.08 | 0.0809 | 6.22 | 0.0136 | 57.1 | <.0001 | 5.97 | 0.0183 | 0.487 | 0.489 |
| 1,7-dimethyluric acid | 1.75 | 0.188 | 0.0976 | 0.755 | 47.6 | <.0001 | 6.31 | 0.0155 | 9.79 | 0.0031 |
| 1-methylxanthine | 9.09 | 0.003 | 1.22 | 0.270 | 40.1 | <.0001 | 1.41 | 0.241 | 2.76 | 0.1038 |
| 3-methylxanthine | 0.847 | 0.359 | 0.163 | 0.687 | 6.38 | 0.0124 | 0.437 | 0.512 | 0.466 | 0.4984 |
| 7-methylxanthine | 4.67 | 0.0321 | 0.0776 | 0.781 | 9.56 | 0.0023 | 0.130 | 0.721 | 0.933 | 0.3394 |
| AAMU | 0.349 | 0.556 | 0.0240 | 0.877 | 31.0 | <.0001 | 4.74 | 0.0345 | 4.91 | 0.032 |

Abbreviations: PD = Parkinson’s disease; PSP = progressive supranuclear palsy; HC = healthy control; AAMU=5-acethylamino-6-amino-3-methyluracil.

*F*-values and *p*-values obtained by analysis of covariance.

**Supplementary Table 2.** **Correlation between caffeine metabolites and disease severity and disease duration in Parkinson’s disease patients in the 2^nd^ cohort.**

|  | H&Y | | MDS-UPDRS part3 | | Disease duration | |
| --- | --- | --- | --- | --- | --- | --- |
| Compound name | ρ | *p*-value | ρ | *p*-value | ρ | *p*-value |
| caffeine | -0.215 | 0.0063 | -0.250 | 0.0015 | -0.272 | 0.0005 |
| theophyline | -0.217 | 0.0058 | -0.241 | 0.0021 | -0.277 | 0.0004 |
| theobromine | -0.129 | 0.104 | -0.108 | 0.176 | -0.142 | 0.0743 |
| paraxanthine | -0.241 | 0.0021 | -0.294 | 0.0002 | -0.273 | 0.0005 |
| 1,3-dimethyluric acid | -0.141 | 0.0752 | -0.131 | 0.0994 | -0.168 | 0.0342 |
| 1,7-dimethyluric acid | -0.181 | 0.022 | -0.193 | 0.0144 | -0.245 | 0.0018 |
| 1-methylxanthine | -0.186 | 0.0185 | -0.185 | 0.0189 | -0.119 | 0.135 |
| 3-methylxanthine | -0.0605 | 0.447 | 0.0076 | 0.924 | -0.0418 | 0.600 |
| 7-methylxanthine | -0.0836 | 0.293 | -0.0299 | 0.708 | -0.0044 | 0.956 |
| AAMU | -0.159 | 0.0453 | -0.149 | 0.0605 | -0.163 | 0.0397 |

Abbreviations: H&Y=Hoehn and Yahr; MDS-UPDRS=Movement Disorders Society-Unified Parkinson's Disease Rating Scale; AAMU=5-acethylamino-6-amino-3-methyluracil.

ρ and *p*-values are obtained by Spearman’s rank correlation coefficient.

**Supplementary Table 3. Association between caffeine metabolites and constipation in Parkinson’s disease patients in the 2^nd^ cohort.**

|  |  |  |  |
| --- | --- | --- | --- |
| Compound name | Constipation | No constipation | *p*-value |
| caffeine | 2.87±0.353 | 3.08±0.561 | 0.681 |
| theophylline | 0.531±0.0435 | 0.598±0.0704 | 0.406 |
| theobromine | 2.29±0.210 | 2.56±0.359 | 0.878 |
| paraxanthine | 2.46±0.194 | 2.80±0.335 | 0.603 |
| 1,3-dimethyluric acid | 0.0291±0.00187 | 0.0306±0.00299 | 0.902 |
| 1,7-dimethyluric acid | 0.0830±0.00704 | 0.0867±0.0102 | 0.678 |
| 1-methylxanthine | 0.0934±0.00737 | 0.0891±0.0100 | 0.759 |
| 3-methylxanthine | 0.171±0.0146 | 0.186±0.0264 | 0.887 |
| 7-methylxanthine | 0.202±0.0158 | 0.230±0.0326 | 0.877 |
| AAMU | 0.545±0.0404 | 0.664±0.0732 | 0.202 |

Abbreviations: PD=Parkinson’s disease; AAMU=5-acethylamino-6-amino-3-methyluracil.

*p*-values are obtained by Wilcoxon test.

**Supplementary Table 4. Correlation between caffeine metabolites and anti-parkinsonian drugs in the 2^nd^ cohort.**

|  | LED | | | | | | levodopa | | | | | |
| --- | --- | --- | --- | --- | --- | --- | --- | --- | --- | --- | --- | --- |
|  | PD | | PSP | | MSA | | PD | | PSP | | MSA | |
| Compound name | ρ | *p*-value | ρ | *p*-value | ρ | *p*-value | ρ | *p*-value | ρ | *p*-value | ρ | *p*-value |
| caffeine | -0.313 | <.0001 | -0.0443 | 0.866 | -0.0443 | 0.866 | -0.244 | 0.0019 | 0.110 | 0.654 | 0.0255 | 0.923 |
| theophylline | -0.295 | 0.0002 | -0.0971 | 0.711 | -0.0971 | 0.711 | -0.217 | 0.0057 | 0.303 | 0.207 | -0.0369 | 0.888 |
| theobromine | -0.153 | 0.0528 | -0.126 | 0.630 | -0.126 | 0.630 | -0.0746 | 0.349 | 0.423 | 0.0710 | -0.149 | 0.569 |
| paraxanthine | -0.306 | <.0001 | -0.0983 | 0.707 | -0.0983 | 0.707 | -0.224 | 0.0045 | 0.320 | 0.181 | -0.0242 | 0.927 |
| 1,3-dimethyluric acid | -0.190 | 0.0162 | -0.111 | 0.671 | -0.039 | 0.882 | -0.104 | 0.189 | 0.251 | 0.300 | 0.0424 | 0.872 |
| 1,7-dimethyluric acid | -0.268 | 0.0006 | -0.0482 | 0.854 | 0.0038 | 0.989 | -0.160 | 0.0439 | 0.070 | 0.775 | 0.0514 | 0.845 |
| 1-methylxanthine | -0.150 | 0.0589 | -0.0390 | 0.882 | -0.0275 | 0.917 | -0.0959 | 0.228 | 0.243 | 0.315 | 0.0013 | 0.996 |
| 3-methylxanthine | -0.0230 | 0.7731 | 0.00380 | 0.989 | -0.111 | 0.671 | 0.0618 | 0.438 | 0.188 | 0.441 | -0.0571 | 0.828 |
| 7-methylxanthine | 0.0055 | 0.945 | -0.0275 | 0.917 | -0.048 | 0.854 | 0.0745 | 0.349 | 0.264 | 0.275 | 0.0331 | 0.900 |
| AAMU | -0.134 | 0.0909 | -0.164 | 0.529 | -0.164 | 0.529 | -0.0504 | 0.526 | -0.0098 | 0.968 | -0.0747 | 0.776 |

Abbreviations: LED=levodopa equivalent dose; PD=Parkinson’s disease; PSP=progressive supranuclear palsy; MSA=multiple system atrophy; AAMU=5-acethylamino-6-amino-3-methyluracil.

ρ and p-values are obtained by Spearman’s rank correlation coefficient.

**Supplementary Table 5. Comparison of *F*-values and *p*-values in each group of the 1^st^ cohort under normalization of age.**

|  | PD vs PSP | | PD vs MSA | | PD vs HC | | PSP vs HC | | MSA vs HC | |
| --- | --- | --- | --- | --- | --- | --- | --- | --- | --- | --- |
|  | F-value | *p*-value | F-value | *p*-value | F-value | *p*-value | F-value | *p*-value | F-value | *p*-value |
| caffeine | 0.411 | 0.5225 | 6.4839 | 0.0121 | 10.9017 | 0.0012 | 11.6759 | 0.0011 | 3.1703 | 0.0799 |
| theophylline | 1.5489 | 0.2154 | 0.1182 | 0.7316 | 11.0992 | 0.0011 | 13.2755 | 0.0005 | 11.5252 | 0.0012 |
| theobromine | 1.0712 | 0.3025 | 1.4714 | 0.2273 | 11.4104 | 0.0009 | 5.888 | 0.0179 | 8.0357 | 0.0062 |
| paraxanthine | 0.7061 | 0.4022 | 0.9282 | 0.3371 | 11.9789 | 0.0007 | 12.8969 | 0.0006 | 12.1878 | 0.0009 |
| 1,7-dimethyluric acid | 0.3392 | 0.5612 | 1.4417 | 0.2321 | 15.7611 | 0.0001 | 12.0196 | 0.0009 | 9.2456 | 0.0035 |
| 1-methylxanthine | 1.905 | 0.1698 | 0.0131 | 0.909 | 31.5549 | <.0001 | 10.7051 | 0.0017 | 12.102 | 0.0009 |
| 3-methylxanthine | 1.2678 | 0.2622 | 2.1967 | 0.1407 | 10.106 | 0.0018 | 4.4851 | 0.0378 | 7.2713 | 0.009 |
| 7-methylxanthine | 2.3577 | 0.127 | 4.759 | 0.0309 | 16.7938 | <.0001 | 7.485 | 0.0079 | 12.2155 | 0.0009 |
| AAMU | 2.309 | 0.1309 | 0.8639 | 0.3544 | 9.2477 | 0.0028 | 17.2756 | <.0001 | 25.2672 | <.0001 |

Abbreviations: PD=Parkinson’s disease; PSP=progressive supranuclear palsy; MSA=multiple system atrophy; AAMU=5-acethylamino-6-amino-3-methyluracil.

*F*-values and *p*-values obtained by analysis of covariance between each pair of groups, using age as covariate.

**Supplementary Table 6. Comparison of *F*-values and *p*-values in each group of the 2^nd^ cohort under normalization of age.**

|  | PD vs PSP | | PD vs MSA | | PD vs HC | | PSP vs HC | | MSA vs HC | |
| --- | --- | --- | --- | --- | --- | --- | --- | --- | --- | --- |
|  | F-value | *p*-value | F-value | *p*-value | F-value | *p*-value | F-value | *p*-value | F-value | *p*-value |
| caffeine | 1.71 | 0.181 | 0.802 | 0.372 | 44.5 | <0.0001 | 3.82 | 0.0568 | 11.1 | 0.0017 |
| theophylline | 1.19 | 0.276 | 0.171 | 0.68 | 46.4 | <0.0001 | 7.05 | 0.0109 | 3.5 | 0.0681 |
| theobromine | 4.17 | 0.0427 | 0.968 | 0.327 | 10.8 | 0.0012 | 0.804 | 0.375 | 1.78 | 0.189 |
| paraxanthine | 7.95 | 0.0054 | 0.0392 | 0.843 | 66.4 | <0.0001 | 5.23 | 0.027 | 10.4 | 0.0024 |
| 1,3-dimethyluric acid | 5.58 | 0.0193 | 1.4 | 0.239 | 65 | <0.0001 | 6.26 | 0.0161 | 1.86 | 0.180 |
| 1,7-dimethyluric acid | 3.01 | 0.0847 | 0.272 | 0.602 | 52.1 | <0.0001 | 6.77 | 0.0125 | 10.8 | 0.002 |
| 1-methylxanthine | 19.5 | <0.0001 | 0.0284 | 0.867 | 43 | <0.0001 | 1.3 | 0.26 | 4.74 | 0.0348 |
| 3-methylxanthine | 2.76 | 0.0983 | 0.297 | 0.587 | 5.38 | 0.0215 | 0.62 | 0.435 | 0.862 | 0.358 |
| 7-methylxanthine | 7.47 | 0.0069 | 0.272 | 0.603 | 9.13 | 0.0029 | 0.273 | 0.604 | 1.64 | 0.207 |
| AAMU | 1.01 | 0.317 | 0.0012 | 0.973 | 33.1 | <0.0001 | 6.24 | 0.0162 | 7.2 | 0.0102 |

Abbreviations: PD=Parkinson’s disease; PSP=progressive supranuclear palsy; MSA=multiple system atrophy; AAMU=5-acethylamino-6-amino-3-methyluracil.

*F*-values and *p*-values obtained by analysis of covariance between each pair of groups, using age as covariate.

**Supplementary Table 7. Correlations of caffeine intake with each metabolite concentration were not significantly different between genders in Parkinson’s disease patients.**

|  | *p*-value | |
| --- | --- | --- |
| Compound name | 1^st^ cohort | 2^nd^ cohort |
| caffeine | 0.759 | 0.574 |
| theophyline | 0.632 | 0.451 |
| theobromine | 0.463 | 0.134 |
| paraxanthine | 0.367 | 0.386 |
| 1,3-dimethyluric acid | - | 0.792 |
| 1,7-dimethyluric acid | 0.653 | 0.572 |
| 1-methylxanthine | 0.337 | 0.344 |
| 3-methylxanthine | 0.561 | 0.236 |
| 7-methylxanthine | 0.737 | 0.281 |
| AAMU | 0.696 | 0.748 |

Abbreviations: AAMU=5-acethylamino-6-amino-3-methyluracil.

*p*-values obtained by logistic regression analysis with daily caffeine intake and gender as covariate.

**Supplementary Table 8. Correlations between daily caffeine intake and each analyte level were not affected by smoking or alcohol habit in Parkinson’s disease patients in the 2^nd^ cohort.**

|  | smoke | alcohol |
| --- | --- | --- |
|  | *p*-value | *p*-value |
| caffeine | 0.223 | 0.780 |
| theophylline | 0.585 | 0.830 |
| theobromine | 0.214 | 0.420 |
| paraxanthine | 0.968 | 0.666 |
| 1,3-dimethyluric acid | 0.420 | 0.784 |
| 1,7-dimethyluric acid | 0.995 | 0.852 |
| 1-methylxanthine | 0.803 | 0.728 |
| 3-methylxanthine | 0.0847 | 0.568 |
| 7-methylxanthine | 0.112 | 0.691 |
| AAMU | 0.0802 | 0.986 |

Abbreviations: AAMU=5-acethylamino-6-amino-3-methyluracil.

*p*-values obtained by logistic regression analysis with daily caffeine intake and current smoker, or habitual alcohol drinker as covariate.

**Supplementary Table 9. Alternative minor allele frequencies of *CYP1A2* and *CYP2E1* variants in both cohorts.**

| **Gene and SNP** | **cDNA** | **Amino acid** | **1^st^ cohort** | | | | ***p*-value** | **2^nd^ cohort** | | | | ***p*-value** | **gnomAD MAF (East Asian)** |
| --- | --- | --- | --- | --- | --- | --- | --- | --- | --- | --- | --- | --- | --- |
|  |  |  | PD | PSP | MSA | HC |  | PD | PSP | MSA | HC |  |  |
| ***CYP1A2*** |  |  |  |  |  |  |  |  |  |  |  |  |  |
| **rs2470890** | c.1612T>C | Asn516Asn | 0.15 | 0.177 | 0.104 | 0.151 | ns | 0.208 | 0.158 | 0.206 | 0.129 | ns | 0.1565 |
| **rs138652540** | c.248C>T | Thr83Met | 0 | 0 | 0 | 0 |  | 0.0089 | 0 | 0 | 0 | ns | 0.0008 |
| **rs35796837** | c.895G>A | Gly299Ser | 0.0136 | 0.0161 | 0 | 0.0116 | ns | 0.003 | 0.0263 | 0 | 0 | ns | 0.0043 |
| **rs45486893** | c.1313C>T | Thr438Ile | 0.0136 | 0 | 0.0208 | 0 | ns | 0.0089 | 0 | 0 | 0.0161 | ns | 0.0035 |
| **rs72547517** | c.1381G>A | Glu461Lys | 0.0091 | 0 | 0 | 0 | ns | 0.0149 | 0 | 0.0294 | 0 | ns | 0.0009 |
| ***CYP2E1*** |  |  |  |  |  |  |  |  |  |  |  |  |  |
| **rs2515641** | c.1296C>T | Phe421Phe | 0.146 | 0.242 | 0.271 | 0.151 | ns | 0.202 | 0.158 | 0.177 | 0.194 | ns | 0.1726 |
| **rs72559710** | c.227G>A | Arg76His | 0 | 0.0161 | 0 | 0 | ns | 0 | 0 | 0 | 0 |  | 0.0043 |
| **rs28371746** | c.909C>A | Thr303Thr | 0.0182 | 0.0323 | 0 | 0.0349 | ns | 0.0268 | 0 | 0 | 0.0323 | ns | 0.0173 |

No significant differences in the frequencies of five SNVs of CYP1A2 and three SNVs of among PD, PSP, MSA, and HC.

Abbreviations: PD = Parkinson’s disease; PSP = progressive supranuclear palsy; MSA = multiple system atrophy; HC = healthy control; SNV: single nucleotide variants; gnomAD = The Genome Aggregation Database; MAF: minor allele frequency; ns = not significant.

^a^*p*-value obtained by chi-square test among all the four groups.

**Supplementary Figure. Metabolic pathways of caffeine and its metabolites measured in the current study**
