# Supplementary figures and images for: Shared Metabolic Profile of Caffeine in Parkinsonian Disorders
Source: Mov Disord. 2020 May 1;35(8):1438–47. doi: 10.1002/mds.28068 (PMC7496239; doi:10.1002/mds.28068)

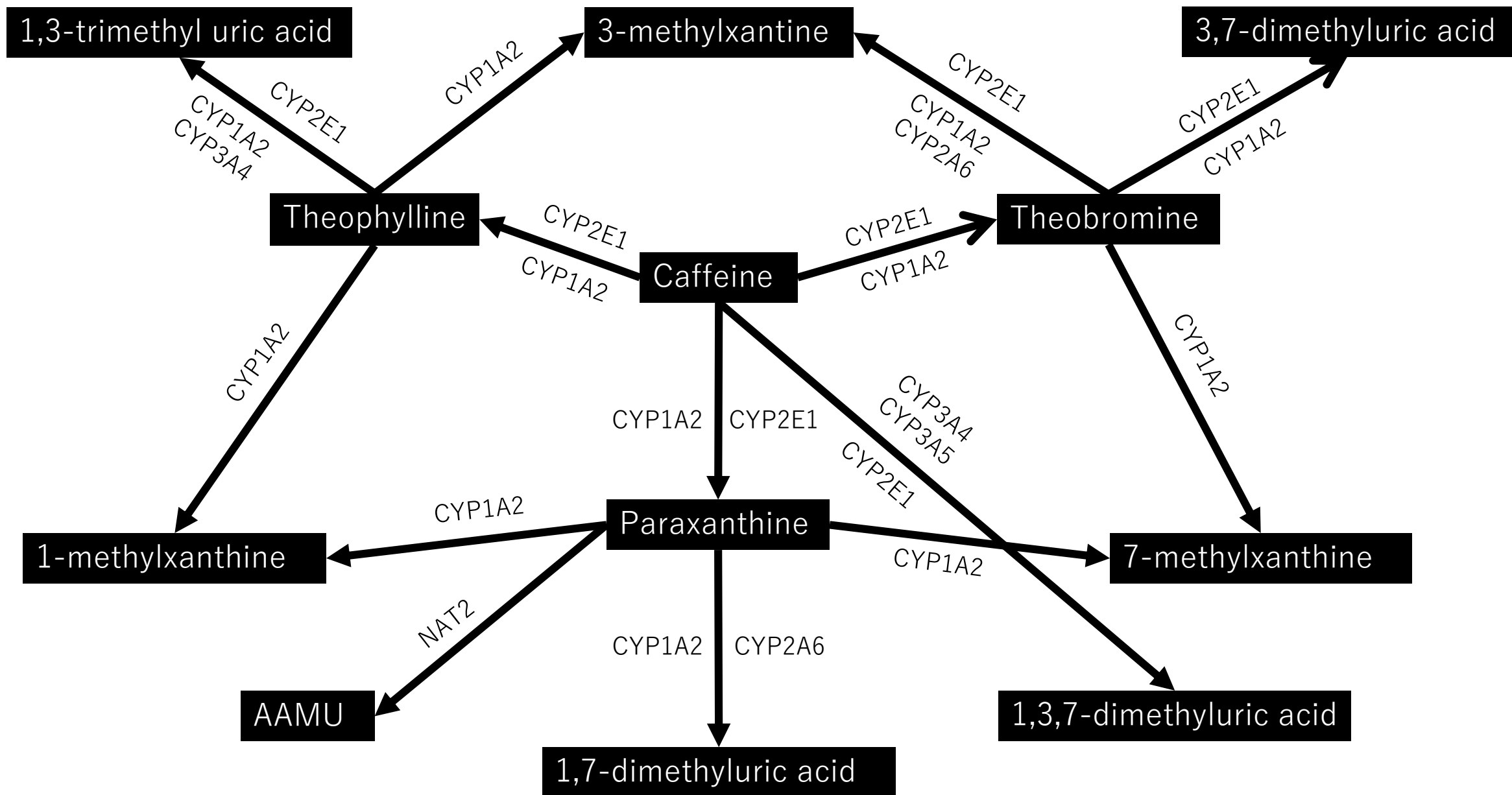

Supplement: Supplementary file 2 — Supplementary Figure. Metabolic pathways of caffeine and its metabolites measured in the current study [file MDS-35-1438-s002.pdf]
